# Supplementary material for: Identification of photoperiod-induced specific miRNAs in the adrenal glands of Sunite sheep (Ovis aries)
Source: Front Vet Sci. 2022 Jul 22;9:888207. doi: 10.3389/fvets.2022.888207 (PMC9354845; doi:10.3389/fvets.2022.888207)
Supplement: Supplementary file 1 [file Data_Sheet_1.ZIP › Supplementary file/miRDeep2 file.zip!/miRDeep2 file/Know_Structure/LP42A1/oar-mir-134.pdf]

miRBase precursor : oar-mir-134
Total read count : 81
oar-miR-134-5p read count : 75
oar-miR-134-3p read count : 6
remaining reads : 0

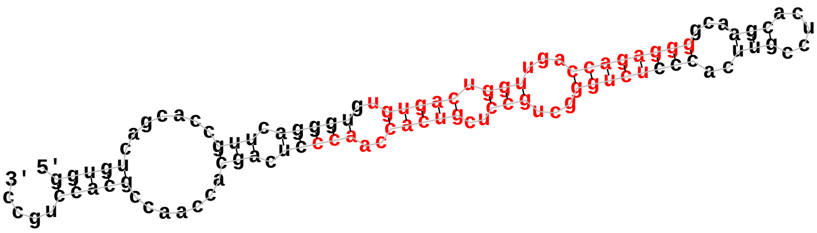

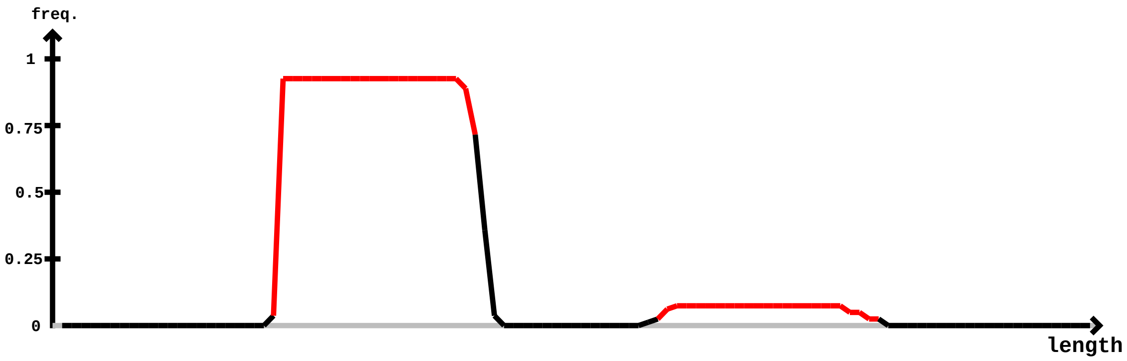


```
                                                                oar-miR-134-3p
                      oar-miR-134-5p
5'- ggugucagcaccguucagggugugugacugguugaccagagggcaagcacuccguucacccucugggcugccucgucaccaacccucagcaccaaccgcaccugcc   -3'    exp
    (((((.......(((.(((((..(((((.(((...((((((((...(((.....)))..))))))))...)))..)))))..))))).))).......)))))....    reads      mm         sample
    ...................gugugacugguugaccagagg...................................................................    3          0          seq
    ......................ugugacugguugaccagag..................................................................    3          0          seq
    ......................ugugacugguugaccagagg.................................................................    11         0          seq
    ......................ugugacugguugaccagaggg................................................................    29         0          seq
    ......................ugugacugguugaccagagggg...............................................................    26         0          seq
    ......................ugugacugguugaccagaggggc..............................................................    3          0          seq
    ............................................................ccucugggcugccucgucaccaa........................    1          0          seq
    .............................................................cucugggcugccucgucaccaa........................    1          0          seq
    ..............................................................ucugggcugccucgucacc..........................    2          0          seq
    ..............................................................ucugggcugccucgucaccaacc......................    1          0          seq
    ...............................................................cugggcugccucgucaccaacc......................    1          0          seq
```
